# Influence of dental occlusion on the athletic performance of young elite rowers: a pilot study

**DOI:** 10.6061/clinics/2017/e453

**Published:** 2018-11-16

**Authors:** Eric Leroux, Stéphanie Leroux, Frédéric Maton, Xavier Ravalec, Olivier Sorel

**Affiliations:** I Service d'Odontologie et Chirurgie Buccale, CHU de Rennes, Rennes, France; II Université de Rennes 1, Rennes, France; III Service de Pédiatrie, CHU de Rennes, Rennes, France; IV Fédération Française d'Aviron, Unité médicale du CREPS de Lille, Wattignies, France

**Keywords:** Posture, Dental Occlusion, Athletic Performance, Malocclusion, Occlusal Splint

## Abstract

**OBJECTIVES:**

The present study aimed to assess the influence of dental occlusion on body posture and the competitive performance of young elite rowers.

**METHOD:**

Dental occlusion disturbance devices were used to simulate dental malocclusions. We assessed the influence of malocclusion on the body balance, paravertebral muscle contraction symmetry, and muscular power of young elite rowers. A nonparametric permutation test for repeated measures ANOVA, a Cochran's Q test for paired data and a paired Student's t-test were used in order to statistically evaluate the influence of artificial occlusal disturbance on each factor. A force platform and a Dyno Concept 2 machine were used as measuring instruments.

**RESULTS:**

A total of 7 members of the “Pôle France Aviron” (age range of 15-17 years) were enrolled in the study. None of the body balance parameters was significantly influenced by the artificial occlusal disturbance. The interposition of an occlusal silicone splint significantly increased the proportion of athletes presenting asymmetric muscular contractions from 14.3% to 85.7% (*p*=0.025) and induced a significant 17.7% decrease in the athletes' muscular power (*p*=0.030).

**CONCLUSIONS:**

This study shows the negative impacts of an occlusal disturbance on the athletic performance of young elite rowers. The detection of malocclusion traits by regular occlusal monitoring would be of great interest in this population.

## INTRODUCTION

Posturology involves studies of the mechanisms of body balance control. In response to motor and sensory controls, the human posture helps maintain body balance in dynamic and static conditions by adapting to environmental requirements. Postural control is usually described as being based on central postural sensors (ocular, mandibular and podal) and peripheral proprioceptive sensors (joints, cutaneous and vestibular sensors) 1. In this postural system, the position of the mandible, assisted by the trigeminal nerve, can both affect and be influenced by posture 2. Influenced by gravity in static conditions, the human body oscillates continuously to maintain balance control 3. The mandibuloacral-spinal postural sensor is involved in the motor control of postural tone. A dental occlusion disturbance stimulates the trigeminal nerve, which induces a muscular and articular chain reaction. Anatomical connections of the trigeminal nerve with the cervical spine, in continuity with the lumbar spine that participates in plantar sensibility, are at the origin of the static postural symptoms described by Clauzade 4,5.

The available literature suggests that in the general population, a dental occlusion disturbance influences vision 6,7, the spatial position of the spine 8, the eccentric force of postural muscles 9-18, the body weight distribution recorded on each foot in a standing position 19,20, and body balance 21-23. A dental occlusion seems to differentially affect postural control depending on static versus dynamic conditions and on whether the eyes are open or closed. The main impact was previously observed to occur in dynamic conditions and with closed eyes 24. Currently, the force platform is the main measuring instrument used for body balance assessment 25-27.

The influence of dental occlusion on body posture seems more pronounced in professional athletes than in the general population 28. The influence of a dental occlusion disturbance on postural stability and competitive athlete performance has already been studied in the context of gun shooting 29, golfing 30 and running 31. Rowing is a discipline of gliding and balance. Coordination is of utmost importance in this comprehensive sport, which mobilizes the bust and the four limbs. This discipline requires optimal postural control for synchronous and symmetrical muscular contractions.

The present study aimed to investigate whether dental occlusion disturbances correlate with alterations in body posture and athletic performance in young elite rowers.

## MATERIALS AND METHODS

### Study design

In the Center of Resources Expertise and Sports Performance (CREPS) of Wattignies (France), we randomized young elite rowers in a prospective, open, randomized controlled crossover trial. A crossover protocol was carried out by performing stabilometric, posturographic and aerobic tests “with then without” or “without then with” artificial occlusal disturbances for groups A and B, respectively. This crossover design was applied in order to reduce the potential learning and/or fatigue bias. The study protocol was approved by the local ethics committee and was in accordance with the recommendations of the Declaration of Helsinki.

### Study participants

We assessed the eligibility of all members of the “Pôle France Aviron” who trained in the CREPS of Wattignies (France) during the inclusion period. To be included, athletes had to meet the following inclusion criteria: 1) age less than 18 years, 2) presence of at least 28 natural or prosthetic teeth in occlusion, 3) absence of alcohol consumption in the last 24 hours preceding the clinical recordings, and 4) affiliation with the social security scheme. The exclusion criteria were as follows: 1) neurological, vestibular or ophthalmic disorders, 2) temporomandibular joint disorders (pain and/or noise), 3) facial symptoms (headache, tinnitus, facial myalgia, bruxism), 4) chronic pain requiring daily drug use for more than three months, 5) pregnancy, or 6) concurrent participation in another study.

Inclusion and exclusion criteria were assessed through a self-questionnaire that was addressed to each athlete. Selected subjects were enrolled in the study after signed informed consent forms were obtained from the subjects and their parents.

### Study interventions

For all participants, a first session (approximately 30 minutes) was dedicated to 1) obtaining maxillary and mandibulary dental arch impressions, 2) manufacturing dental plaster casts from these dental impressions, 3) assembling these plaster molds on a semi-adjustable articulator (Sam 2 system), and 4) manufacturing 3 unilateral occlusal disturbance devices (two silicone splints increasing the vertical dimension of occlusions by 1 and 2 millimeters and a silicone splint inducing a 4-millimeter lateral deflection of the mandible). The second session (approximately 50 minutes) was then conducted according to the three steps described below.

#### Step 1: Evaluation of the impact of the artificial occlusal disturbance on the athlete's body balance – stabilometric tests

Body balance tests were performed using a 5-Hz sampling frequency force platform, which met the metrological requirements of the French Posturology Association (Cyber-Sabots^TM^, SABOSOFT software, France). Each test lasted 51.2 seconds as recommended by the French Posturology Association (AFP-85 Standards). All recordings were carried out in the following standardized conditions 32: the force plate was placed perpendicularly, 150 cm from a wall; the subjects were required to remain as stable as possible, relaxed, with arms hanging free beside their trunks, and facing the wall; the placement of the subjects on the force plate was standardized, with a foot position in accordance with the AFP-85 recommendations, namely, an angle of divergence of 30° and a distance between the heels of 2 cm; and quiet conditions were maintained during the exam, and disturbing elements were eliminated to avoid any influence on posture.

At this step, participants performed 10 force platform tests, and they had to maintain a standing position under the following dental occlusion conditions.

- Without an artificial occlusal disturbance: Maximal Intercuspal Occlusion (MIO, i.e., with the maximum number of teeth in contact, mouth closed), with eyes open (EO) or eyes closed (EC).
- With an artificial occlusal disturbance: MIO with a silicone splint increasing the vertical dimension of the occlusion by 1 millimeter, with EO or EC; MIO with a silicone splint increasing the vertical dimension of the occlusion by 2 millimeters, with EO or EC; and MIO with a silicone splint resulting in a 4-millimeter lateral deflection of the mandible, with EO or EC.

This sequence of 10 tests was performed twice to verify the reproducibility of the results. A resting period of 1 minute was observed between each recording to avoid muscular fatigue effects 24. During each test, three stabilometric parameters were measured: the projected sway area (i.e., the projection of the center of foot pressure displacement, expressed in mm^2^), sway velocity (i.e., the average speed of the center of foot pressure, expressed in mm/s) and velocity variance.

#### Step 2: Evaluation of the impact of the artificial occlusal disturbance on the symmetry of the athlete's muscular contraction – posturographic tests

This step comprised an assessment of the symmetry of paravertebral muscular contractions during the posterior-superior iliac spine (PSIS) test, according to dental occlusion conditions. The PSIS test was performed in the following standardized conditions: the participant stood on a foam mat (deletion of the podal postural sensor) stretching the legs and with MIO; the operator placed his thumbs on the participant's posterior-superior iliac spines; the participant rolled over on his own, bending his head and bust to the maximum without bending legs. This PSIS test was performed with EO, in the MIO position, under the following dental occlusions conditions: 1) without artificial occlusal disturbance and 2) with interposition of a silicone splint resulting in a 4-millimeter lateral deflection of the mandible.

**Figure 1:**
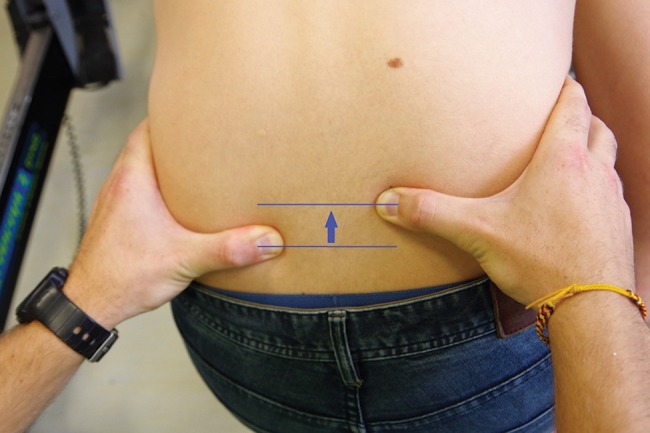
Abnormal response to the posterior-superior iliac spine test: asymmetric contractions of the paravertebral muscles.

This sequence of the two tests was performed twice to verify the reproducibility of the results. During this test, the endpoint was qualitative: the operator's thumbs' ascension reflected whether the contractions of the paravertebral muscles were asymmetric (abnormal response) (Figure 1) or not (normal response).

#### Step 3: Evaluation of the impact of the artificial occlusal disturbance on the athlete's muscular power – aerobic tests

The muscular power of the athletes was evaluated with the “leg press” test on the “Dyno Concept 2” machine. This “leg press” test is a standardized and validated aerobic test, which is especially suitable for rowing 33. The following standardized conditions were met: each leg press test was performed after a standardized muscular warm-up (defined with the coach); each test lasted 30 seconds; and the participant sat on a fixed seat, with feet placed on the flexfoot, with EO, the bust remaining motionless and the hands holding the handles located under the seat. The test consisted of performing extension/flexion movements of the legs.

This leg press test was performed with EO, in the MIO position, under the following dental occlusion conditions: 1) without artificial occlusal disturbance, 2) with interposition of a silicone splint resulting in a 4-millimeter lateral deflection of the mandible.

This sequence of 2 tests was performed twice to verify the reproducibility of the results. A resting period of 1 minute was observed between each recording to avoid muscular fatigue effects 24. During this test, the endpoint was the measure of muscular power, expressed in watts.

At each step, test order (“with then without” or “without then with” artificial occlusal disturbance) was determined by the randomization group. All measurements were recorded by the same operator.

### Study outcomes

The primary outcome was the muscular power developed by the athletes during the “leg press” aerobic test.

The values of the stabilometric parameters (sway area, sway velocity, and velocity variance) and the symmetry of the paravertebral muscle contractions during the PSIS test were assessed as secondary endpoints.

### Sample size and statistical analysis

The sample size (11 subjects in each group) was calculated based on an expected 15% decrease in muscular power in the presence of an artificial occlusal disturbance, a level of significance of 5% and a power of 90%.

The paired Student's t-test was used to assess the relationship between the dental occlusion condition (with or without an artificial occlusal disturbance) and the muscular power of the young rowers. Normality of the distribution was previously verified with the Shapiro-Wilk test. Regarding secondary endpoints, a nonparametric permutation test for repeated measures ANOVA was performed to evaluate the influence of visual and occlusal conditions on sway area, sway velocity and velocity variance. A Cochran's Q test for paired data was used to evaluate the relationship between dental occlusion conditions and muscular contraction symmetry. Statistical significance was indicated by a *p*-value<0.05. All analyses were conducted using R (version 3. 4.1).

## RESULTS

### Study participants

**Figure 2:**
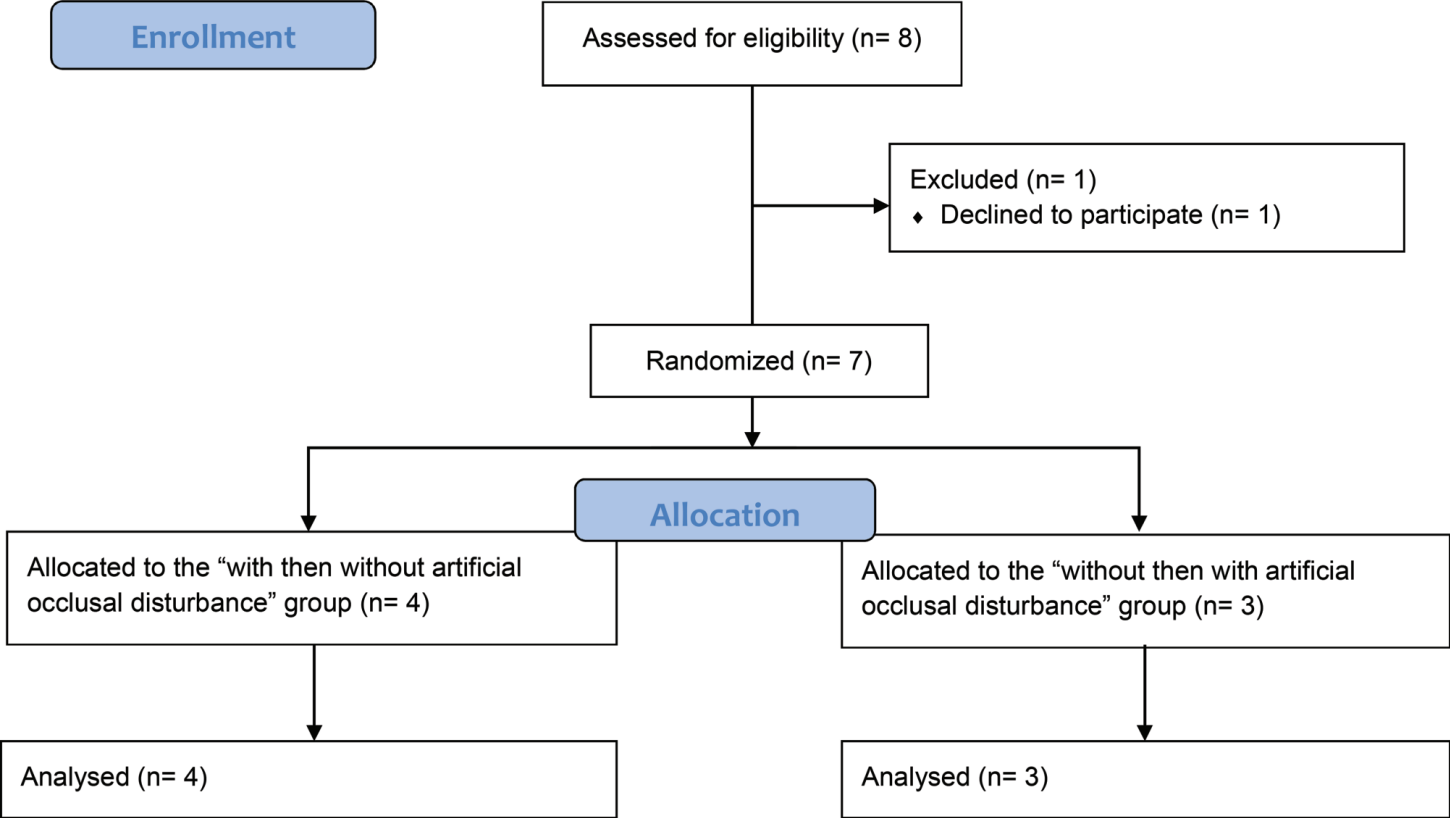
Study flowchart.

Of the 8 members of the “Pôle France Aviron” that trained in the CREPS of Wattignies (France) during the inclusion period, all met the study eligibility criteria, but one athlete declined to participate. Therefore, between September and October 2017, a total of 7 subjects were enrolled in the study (Figure 2). The population's characteristics are detailed in Table 1. The median weight and height were 73 kg and 186 cm, respectively.

### Impact of the artificial occlusal disturbance on the athlete's body balance

Tables 2 and 3 present the results of the body balance parameter analysis.

With all occlusal conditions combined, closing of the eyes significantly increased the sway area, sway velocity and velocity variance, with median values increasing from 285.8 to 482.1 mm^2^ (*p*=0.002), from 8.7 to 13.2 mm/s (*p*=0.001) and from 40.2 to 104.3 (*p*=0.001), respectively (Table 3). However, none of the three body balance parameters was significantly influenced by the artificial occlusal disturbance. We only observed a greater dispersion of the parameters' values when the lateral silicone splint was present. In the same EC condition, the interquartile range value of the sway area was 1.5 times larger with the artificial 4-mm mandibular laterodeviation than without (601.5 *versus* 389.8) (Table 2).

### Impact of the artificial occlusal disturbance on the symmetry of the athlete's muscular contractions

Table 4 presents the proportion of subjects presenting abnormal responses to the PSIS test according to the presence/absence of an artificial occlusal disturbance. The interposition of the silicone splint resulting in a 4-millimeter lateral deflection of the mandible increased the proportion of asymmetric muscular contractions from 14.3% to 85.7% of the participants (*p=*0.025). An abnormal ascension of the operator's thumb was always observed from the ipsilateral side of the silicone splint.

The artificial occlusal disturbance did not modify the response to the PSIS test in two subjects. One of them already presented an abnormal response without the silicone splint; the other athlete still presented a normal response with the occlusal disturbance.

### Impact of the artificial occlusal disturbance on the athlete's muscular power

The mean value of the muscular power obtained in the presence of the artificial occlusal disturbance was 498.2 watts (standard deviation 202.5), which was 107 watts lower than the value obtained without the artificial occlusal disturbance (mean value 605.3 watts, standard deviation 244.7). The interposition of the silicone splint resulting in a 4-millimeter lateral deflection of the mandible induced a significant 17.7% reduction in the athletes' muscular power (*p*=0.030). Normality of the muscular power distribution was previously verified (*p*=0.924).

## DISCUSSION

To our knowledge, this is the first study evaluating the impact of dental occlusions on athletic performance in rowing. Using artificial occlusal disturbances, we showed a significant negative impact of malocclusion on the posture and muscular power of young elite rowers.

By evaluating the impact of an artificial occlusal disturbance on the athletes' body balance, vision was shown to significantly affect the sway area, sway velocity and velocity variance. A loss of postural control was observed when rowers closed their eyes, as demonstrated by an increase in the three postural parameter values. The suppression of the ocular sensor (main postural sensor) induced by the closed eye condition explains this result. However, we did not observe significant effects of the mandibular sensor disturbance on the three body balance parameters. This could be explained by the small study population size, certainly inducing a lack of statistical power. We can also suppose that an occlusal disturbance influences body balance, only after a prolonged time of neuronal integration 4,5; this was not assessed during our one-hour test conditions. Contradictory results have been reported on potential correlations between dental occlusion and body posture. Our findings are in accordance with previous studies by Tardieu et al., Perinetti et al. and Baldini et al. 24,26,27,32 and with recent results by Rocha et al. 34. One author found a significant relationship between the mandibular position and sway area in an older age group from the general population (age range 21-53 years) 2.

Regarding the results of the PSIS test, the artificial lateral deflection of the mandible induced an asymmetric contraction of the lumbar erector spinae muscles in 85.7% of the athletes. This finding is in accordance with the mechanism described by Clauzade. The stimulation of the trigeminal nerve by the artificial occlusal disturbance generates a muscular and articular chain reaction, including inclination and rotation of the occiput on the first cervical vertebra, inclination and rotation of the cervical spine, ascent of the ipsilateral scapula, and ascent of the ipsilateral posterior-superior iliac spine 4,5. In the same way, using rasterstereography, März et al. recently demonstrated significant alterations in the fleche lombaire in different dental occlusion conditions 35. The two subjects manifesting unexpected responses to the PSIS test presented atypical physical conditions. One of them was affected by scoliosis, an abnormal pelvic rotation and leg length inequality, which could explain the abnormal response to the PSIS test already observed before the interposition of the silicone splint. The other was treated with orthodontic appliances at the time of the study.

At the last step, the interposition of the silicone splint significantly altered the athletes' muscular power during the leg press test. To date, only one study has provided data on muscle power measured during a leg press test on a “Dyno Concept 2” 33 machine, and the influence of the occlusal disturbance on this parameter was never studied. We hypothesize that the muscular power alteration of the rower is a consequence of a postural control perturbation due to asymmetrical and asynchronous muscular contractions in the presence of an artificial occlusal disturbance. Similarly, in a recent study by Grosdent et al., dental occlusal disturbance immediately induced significant alterations in eccentric quadricep and hamstring performances 36. Future studies should analyze the immediate effect of an artificial occlusal disturbance on the electromyographic (EMG) activity of the main postural muscles used by rowers, such as the masseter, sternocleidomastoid, biceps, erector spinae, quadriceps and soleus muscles. The use of mandibular repositioning splints in rowers with occlusal disorders can be an interesting therapeutic approach to optimizing their neuromuscular coordination and competitive performances.

In this pilot study, artificial mandibular laterodeviation induced a significant alteration in the muscular power of the rowers. Such temporomandibular disorders constitute a major public health problem 37. Based on our findings, dental occlusion examination should be regularly undertaken for young elite rowers. Moreover, for cases in which dental malocclusions are detected, a suitable treatment plan based on prosthetic, surgical and/or orthodontic care can improve athletes' performances.

Due to the elite rowers' imposed training schedule, one limitation of this study was that we were unable to respect a strict 24-hour rest period before the study recordings. The second limitation was the small number of participants. Our preliminary results must now be confirmed by larger studies.

Within the limitations of this pilot study, we demonstrated the negative impact of occlusal disturbance on the athletic performances of young elite rowers. The detection of malocclusion traits by regular occlusal monitoring would be of utmost interest in this population.

## AUTHOR CONTRIBUTIONS

Leroux E, Leroux S and Maton F designed the study and performed the experiment. Ravalec X and Sorel O coordinated the study.

## Figures and Tables

**Table 1:** Population characteristics.

Variable	Number	Median (range)
Sex (Female/Male)	3/4	
Age		16 (15-17)
Weight (kg)		73 (72-103)
Height (cm)		186 (167-205)

**Table 2:** Distribution of body balance parameters according to occlusal and eye conditions.

Parameter (unit)	Occlusal condition^1^	Eye condition	Median	IQR^2^
Sway Area	MIO	Eyes open	304.0	253.9
(mm^2^)	MIO	Eyes closed	496.9	389.8
	MIO with 1-mm increased VDO	Eyes open	267.7	243.9
	MIO with 1-mm increased VDO	Eyes closed	414.9	226.6
	MIO with 2-mm increased VDO	Eyes open	409.3	412.1
	MIO with 2-mm increased VDO	Eyes closed	583.5	317.1
	MIO with 4-mm lateral deflection	Eyes open	201.0	458.2
	MIO with 4-mm lateral deflection	Eyes closed	408.8	601.5
Sway Velocity	MIO	Eyes open	9.0	5.5
(mm/s)	MIO	Eyes closed	13.51	5.6
	MIO with 1-mm increased VDO	Eyes open	7.8	5.1
	MIO with 1-mm increased VDO	Eyes closed	12.9	5.2
	MIO with 2-mm increased VDO	Eyes open	9.2	4.4
	MIO with 2-mm increased VDO	Eyes closed	15.1	4.9
	MIO with 4-mm lateral deflection	Eyes open	8.5	6.3
	MIO with 4-mm lateral deflection	Eyes closed	12.6	7.7
Velocity Variance	MIO	Eyes open	45.8	46.0
	MIO	Eyes closed	98.6	83.4
	MIO with 1-mm increased VDO	Eyes open	33.1	51.7
	MIO with 1-mm increased VDO	Eyes closed	71.8	90.3
	MIO with 2-mm increased VDO	Eyes open	45.6	91.9
	MIO with 2-mm increased VDO	Eyes closed	123.4	58.2
	MIO with 4-mm lateral deflection	Eyes open	34.8	56.2
	MIO with 4-mm lateral deflection	Eyes closed	73.3	204.5

^1^MIO: Maximal Intercuspal Occlusion; increased VDO: silicone splint increasing the vertical dimension of occlusion; lateral deflection: silicone splint resulting in a lateral deflection of the mandible.

^2^IQR: Interquartile Range.

**Table 3:** ANOVA results for the impact of the artificial occlusal disturbance on the athlete’s body balance.

Parameter	Influence^1^	*p*-value^2^
Sway area (mm^^2^^)	Eye condition	0.002*
	Occlusal condition	0.197
Sway velocity (mm/s)	Eye condition	0.001*
	Occlusal condition	0.466
Velocity variance	Eye condition	0.001*
	Occlusal condition	0.550

^1^Eye condition: open eyes/closed eyes.

Occlusal condition: Maximal Intercuspal Occlusion (MIO)/MIO with a silicone splint increasing the vertical dimension of the occlusion by 1 millimeter/MIO with a silicone splint increasing the vertical dimension of the occlusion by 2 millimeters/MIO with a silicone splint resulting in a 4-millimeter lateral deflection of the mandible.

^2^*p*-values of the permutation test for repeated measures ANOVA.

**Table 4:** Impact of the artificial occlusal disturbance on the response to the posterior-superior iliac spine (PSIS) test.

Occlusal condition^1^	Normal response to PSIS test (N subjects)	Abnormal response to PSIS test (N subjects)
MIO	6	1
MIO with 4-mm lateral deflection	1	6

1MIO: Maximal Intercuspal Occlusion; lateral deflection: silicone splint resulting in a lateral deflection of the mandible.
